# Supplementary material for: Clinician motivational interviewing skills in ‘simulated’ and ‘real-life’ consultations differ and show predictive validity for ‘real life’ client change talk under differing integrity thresholds
Source: PeerJ. 2023 Oct 2;11:e14634. doi: 10.7717/peerj.14634 (PMC10552748; doi:10.7717/peerj.14634)
Supplement: Supplemental Information 1 [file peerj-11-14634-s001.docx]

| Variable | Interpretation |
| --- | --- |
| Frequency Counts of Behaviour | |
| Giving Information | Gives information, educates, provides feedback, or expresses a professional opinion without persuading, advising, or warning (i.e. does not imply the information is specifically relevant to the client or that the client must act on it) |
| Persuade | Overt attempts to change a client’s opinions, attitudes or behaviours using tools such as logic, compelling arguments, self-disclosure, facts, biased information, advice, suggestions, tips, opinions, or solutions to problems |
| Persuade with Permission | Emphasis on collaboration or autonomy support while using persuasion |
| Question | Open or closed |
| Simple Reflection | Repeats, re-phrases or paraphrases the client’s previous statement adding little or no meaning or emphasis to what the client has said |
| Complex Reflection | Repeats, re-phrases or paraphrases the client’s previous statement adding substantial meaning or emphasis to what the client has said |
| Affirm | States something positive about the client’s strengths, efforts, intentions or worth |
| Seeking Collaboration | Explicitly attempts to share power or acknowledge the expertise of the client |
| Emphasise Autonomy | Highlights a client’s sense of control, freedom of choice or self-direction over change |
| Confront | Directly and unambiguously disagreeing, arguing, correcting, shaming, blaming, criticizing, labeling, warning, moralizing, ridiculing or questioning a client’s honesty |
|  |  |
| Global Scores (Scale 1-5) | |
| Cultivating Change Talk | Encourages the client’s own language in favor of the change goal and confidence for making that change |
| Softening Sustain Talk | Avoids a focus on the reasons against changing or on maintaining the status quo |
| Partnership | Conveys an understanding that expertise and wisdom about change reside mostly within the client and actively fosters collaboration and power-sharing |
| Empathy | Understands or makes an effort to grasp the client’s perspective and experience. Reflective listening is an important part of this characteristic, but it encompasses *all* attempts made to understand the client and actively communicate this understanding |
| Summary Measurements | |
| Relational | (Partnership + Empathy)/2 |
| Technical | (Cultivating Change Talk + Softening Sustain Talk)/2 |
| Reflection to question ratio | (Simple Reflection + Complex Reflection)/Total Question |
| Percent complex Reflection | Complex Reflection/(Simple Reflection + Complex Reflection) |
| MI-adherent | Affirmation + Emphasise Autonomy + Seeking Collaboration |
| MI Non-adherent | Persuade + Confront |
