# Supplementary material for: Clinician motivational interviewing skills in ‘simulated’ and ‘real-life’ consultations differ and show predictive validity for ‘real life’ client change talk under differing integrity thresholds
Source: PeerJ. 2023 Oct 2;11:e14634. doi: 10.7717/peerj.14634 (PMC10552748; doi:10.7717/peerj.14634)
Supplement: Supplemental Information 2 [file peerj-11-14634-s002.docx]

| Variable | Description |
| --- | --- |
| Change Talk | |
| Reason  Subcode: desire  Subcode: ability  Subcode: need | Specific rationale, basis, incentive or justification for making the change |
|  | As above, containing words ‘want’, ‘desire’, ‘like’ or close synonym |
|  | As above, containing words ‘can’, ‘possible’, ‘willpower’ or close synonym |
|  | As above, containing words ‘need’ or ‘must’ or close synonym |
| Taking steps | Concrete action(s) towards the change |
| Commitment | Agreement, intention or obligation towards the change |
| Other | Any language that clearly reflects movement towards change that is not captured by the other categories |
| Neutral | |
| Follow/neutral | No indication of farmer inclination either towards or away from change |
| Sustain Talk | |
| Reason  Subcode: desire  Subcode: ability  Subcode: need | Specific rationale, basis, incentive or justification away from making the change or to maintain the status quo  As above, containing words ‘want’, ‘desire’, ‘like’ or close synonym  As above, containing words ‘can’, ‘possible’, ‘willpower’ or close synonym  As above, containing the words ‘need’ or ‘must’ or close synonym |
| Taking Steps | Concrete actions away from the change or to maintain the status quo |
| Commitment | Agreement, intention or obligation away from the change or to maintain the status quo |
| Other | Any language that clearly reflects movement away from the change or to maintain the status quo that is not captured by the other categories |
