# Supplementary material for: Clinician motivational interviewing skills in ‘simulated’ and ‘real-life’ consultations differ and show predictive validity for ‘real life’ client change talk under differing integrity thresholds
Source: PeerJ. 2023 Oct 2;11:e14634. doi: 10.7717/peerj.14634 (PMC10552748; doi:10.7717/peerj.14634)
Supplement: Supplemental Information 3 — Note. MITI = Motivational Interviewing Treatment Integrity coding system 4.2.1 (Moyers, Manuel & Ernst, 2014); Global Score = Likert data 1–5; Verbal Behaviour = continuous data (frequency count) a Indicates data where Cohort B diminish in MI skills from Simulated Interaction to Real Life that is incongruent with Cohort A [file peerj-11-14634-s003.docx]

|  |  |  | Cohort A | | |  | |  | | | Cohort B | |  |
| --- | --- | --- | --- | --- | --- | --- | --- | --- | --- | --- | --- | --- | --- |
| MITI component | MI skill | SI | | | RL | | | | SI | | | RL | |
|  |  | *M* | | *SD* | *M* | | *SD* | | *M* | *SD* | | *M* | *SD* |
| Global Score | Cultivating Change Talk | 2.49 | | 0.78 | 1.44 | | 0.67 | | 2.79 | 0.77 | | 1.76 | 0.79 |
|  | Softening Sustain Talk | 3.84 | | 0.37 | 3.09 | | 0.70 | | 3.34 | 0.65 | | 3.48 | 0.67 |
|  | Partnership | 2.76 | | 0.95 | 1.74 | | 0.81 | | 3.51 | 0.75 | | 2.42 | 0.83 |
|  | Empathy | 2.53 | | 0.92 | 1.89 | | 0.86 | | 3.40 | 0.79 | | 2.73 | 0.93 |
| Verbal Behaviour | Giving Information | 15.84 | | 6.20 | 20.07 | | 8.31 | | 9.25 | 2.77 | | 13.97 | 6.83 |
|  | Persuade | 9.22 | | 5.71 | 6.15 | | 4.97 | | 3.62 | 3.38 | | 4.40 | 2.74 |
|  | Persuade with Permission ^a^ | 1.53 | | 1.43 | 0.93 | | 1.36 | | 5.26 | 3.00 | | 1.11 | 1.47 |
|  | Question | 8.69 | | 5.98 | 13.59 | | 9.50 | | 11.19 | 5.00 | | 17.97 | 11.30 |
|  | Simple Reflection | 2.41 | | 1.58 | 5.25 | | 4.38 | | 2.74 | 2.10 | | 6.36 | 5.96 |
|  | Complex Reflection | 2.31 | | 2.01 | 2.48 | | 2.75 | | 4.02 | 2.71 | | 6.98 | 4.85 |
|  | Affirm | 1.39 | | 1.52 | 2.54 | | 2.92 | | 2.09 | 1.96 | | 2.65 | 1.99 |
|  | Seeking Collaboration | 2.51 | | 1.84 | 0.45 | | 0.82 | | 3.21 | 2.06 | | 0.83 | 1.12 |
|  | Emphasise Autonomy | 0.22 | | 0.54 | 0.09 | | 0.40 | | 0.42 | 0.66 | | 0.14 | 0.39 |
|  | Confront | 0.25 | | 0.72 | 0.13 | | 0.44 | | 0.19 | 0.14 | | 0.09 | 0.33 |
